# Combined Effects of Circulating Levels of 25-Hydroxyvitamin D and Th1 and Th2 Cytokines on Breast Cancer Estrogen Receptor Status

**DOI:** 10.3390/cancers6010211

**Published:** 2014-01-27

**Authors:** Song Yao, Chi-Chen Hong, Susan E. McCann, Gary Zirpoli, Lei Quan, Zhihong Gong, Candace S. Johnson, Donald L. Trump, Christine B. Ambrosone

**Affiliations:** 1 Department of Cancer Prevention and Control, Roswell Park Cancer Institute, Buffalo, NY 14263, USA; E-Mails: chi-chen.hong@roswellpark.org (C.-C.H.); susan.mccann@roswellpark.org (S.E.M.); gary.zirpoli@roswellpark.org (G.Z.); lei.quan@roswellpark.org (L.Q.); zhihong.gong@roswellpark.org (Z.G.); christine.ambrosone@roswellpark.org (C.B.A.); 2 Department of Pharmacology and Therapeutics, Roswell Park Cancer Institute, Buffalo, NY 14263, USA; E-Mail: candace.johnson@roswellpark.org; 3 Department of Medicine; Roswell Park Cancer Institute, Buffalo, NY 14263, USA; E-Mail: donald.trump@roswellpark.org

**Keywords:** interactions, 25-hydroxyvitamin D, cytokines, breast cancer, epidemiology, biomarker

## Abstract

Vitamin D has been recognized for its immune-modulating properties. We have previously found that levels of 25OHD, and cytokines including IL5, IFNα2, and TNFα, are also associated with estrogen receptor (ER) negative breast cancer in younger women. Thus, we hypothesized that there may be interactions between vitamin D and the immune system in influencing breast cancer ER status, which was tested in 490 women with incident breast cancer. There was no correlation of the levels of 25OHD with any cytokine, and their associations with tumor ER negative status were independent of each other. However, premenopausal women with low 25OHD and high TNFα levels had the highest likelihood of having ER negative cancer (odds ratio [OR] = 7.32, 95% confidence interval [CI] = 2.44−21.98), with evidence of synergy between the two (relative excess risk due to interaction [RERI] = 5.46, p for additive interaction = 0.14, and p for multiplicative interaction = 0.09). There were similar synergistic associations between 25OHD and IL5, and several IFNα2 to Th2 cytokine ratios. This is the first study to provide evidence of interactions between vitamin D and the immune system in relation to breast cancer ER status, which may inform combinational use of vitamin D and anti-inflammatory drugs for cancer prevention and therapy.

## 1. Introduction

There is a rich body of evidence supporting vitamin D as a potent immunomodulator [1,2,3,4]. VDR and vitamin D activating enzyme 1α-hydroxylase is expressed in almost all immune cells [5]. While enhancing innate immune responses against bacterial infection, vitamin D modulates adaptive immunity by influencing the actions of antigen presenting cells (APCs) and T-helper lymphocytes. Consistent with its roles in mediating tolerant immunity, vitamin D has been linked to a number of autoimmune diseases [6], and vitamin D supplementation is efficacious in preventing or curing autoimmune diseases in some preclinical studies [1]. Among the genes upregulated by vitamin D stimulation, it was found that genes involved in autoimmune diseases are particularly enriched [7]. Moreover, in randomized clinical trials of patients with cardiovascular disease and colorectal adenoma, vitamin D supplementation significantly alters patients’ immune profiles [8,9].

Given the evidence of potential immuno-modulating activities of vitamin D, it is plausible to speculate that there may be interactions between vitamin D and the immune system relevant to the risk of breast cancer. It has been shown in prostate cancer cell lines that the combination of calcitriol with the non-steroidal anti-inflammatory drugs (NSAIDs), including naproxen and ibuprofen, can achieve the same magnitude of cancer cell growth inhibition at 1/2–1/10 the concentrations of the drugs used as a single agent [10]. Similar synergistic effects have not yet been examined in breast cancer. 

A number of epidemiologic studies have examined the role of vitamin D in breast cancer etiology with inconclusive results. One reason for the inconsistent findings might be cancer heterogeneity—the association of a risk factor with cancer may differ by cancer subtypes. For breast cancer, estrogen receptor (ER) is one of a few most important prognostic and predictive biomarkers for breast cancer. It is likely that ER-positive and ER-negative breast cancer follows distinct etiology pathways. We hypothesized that vitamin D is related to breast cancer in an ER-status specific way, which were examined by comparing ER+ *vs.* ER− cancer. In our previous study, we found that high blood levels of 25-hydroxyvitamin D (25OHD) were associated with a lower risk of breast cancer, particularly risk of the estrogen receptor (ER)-negative and triple-negative cancer subtype, in premenopausal women [11]. Similarly to 25OHD, we subsequently found that high levels of plasma cytokines, including interferon α2 (IFNα2), tumor necrosis factor α (TNFα), and interleukin 5 (IL5), as well as several ratios of T-helper type 1 (Th1) to Th2 cytokines, were associated with increased likelihood of ER negative and triple-negative breast cancer compared to ER positive or luminal A tumors in premenopausal women [12]. Although a few cytokines have been examined in relation to ER- and triple-negative breast cancer, these studies focused mostly on tumor-associated changes in the microenvironment. Our study was the largest examining circulating levels of a variety of cytokines, chemokines and their receptors. Given these previous results and the established literature on the immuno-modulating properties of vitamin D [5], we speculated that the associations of 25OHD and cytokines with breast cancer characteristics may be inter-correlated and that the combinational associations are stronger than either factor alone.

Among 490 women, newly-diagnosed with invasive breast cancer in our previous studies of 25OHD and cytokines, we examined the inter-dependence of the associations of 25OHD and cytokines and further, their combinational associations with breast cancer ER status.

## 2. Patient Population and Methods

### 2.1. Patient Population

Detailed description of the patient population has been published elsewhere [11,12]. Briefly, data and specimens from women with invasive breast cancer were obtained from the Data Bank and Biorepository (DBBR) at Roswell Park Cancer Institute (RPCI). DBBR is a comprehensive data and sample bank containing pretreatment biospecimens that are rigorously collected and processed, with comprehensive clinical and epidemiologic data [13]. These analyses were performed using data from the 490 women self-identified as non-Hispanic white, diagnosed at RPCI with histologically confirmed primary, first-time invasive breast cancer, and with no prior cancer history except non-melanoma skin. They were all breast cancer patients diagnosed between December 2003 to June 2009 and from whom both serum 25OHD and selected plasma cytokines levels were measured. Patients’ clinical data, including tumor stage, histologic grade and estrogen receptor (ER) status, as determined by standard clinical immunohistochemical assays, were obtained from a clinical database maintained by the RPCI breast program and supplemented with data from abstracted medical records and the RPCI Tumor Registry. Because of the limited number of triple-negative cancer cases, particularly after stratification by menopausal status, triple-negative status was not analyzed in this study. Self-administered questionnaires were used to collect data on demographics, reproductive factors, medical history, family history of cancer, and lifestyle factors. Postmenopausal status in the study was defined as women who experienced 12 consecutive months of amenorrhea, or women who underwent bilateral salpingo-oophorectomy. All patients signed the informed consent for DBBR sample banking, and the study was approved by the Institutional Review Board at RPCI.

### 2.2. Blood Samples, Serum 25-Hydroxyvitamin D Assay, and Plasma Cytokine Assays

Blood samples were collected, prior to surgery and any adjuvant treatment, in phlebotomy when specimens for clinical measures are drawn, transported to the laboratory through a pneumatic tube system, and processed within one hour of blood draw. Specimens were maintained in liquid nitrogen until analysis. Serum 25-hydroxyvitamin D (25OHD) concentrations were measured by the immunochemiluminometric assay on the DiaSorin Liasion automated instrument performed by Heartland Assays (Ames, IA, USA). The coefficient of variation (CV) from laboratory technical QCs was 8.8%. A panel of 27 cytokines was measured based on plasma samples by Luminex xMAP immune-bead array assays (Millipore, Bellerica, MA, USA) performed by the RPCI Flow Cytometry Core. Intra-plate CVs from laboratory technical QCs ranged from 1.4% to 7.5% and inter-plate CVs ranged from 2.7% to 11.9%. To reduce the burden of multiple testing, only cytokines and ratios which had been significantly associated with breast cancer ER status in our previous study were included in this analysis; these were IL5, IFNα2, TNFα, six IFNα2 to Th2 cytokines ratios (IFNα2/IL4, IFNα2/IL10, IFNα2/CCL2, IFNα2/CCL7, IFNα2/CCL11, IFNα2/TNFα), and four Th1 cytokine to IL5 ratios (IL12p70/IL5, IFNγ/IL5, CXCL10/IL5, and TNFα/IL5). 

### 2.3. Statistical Analysis

Descriptive characteristics of the patient population were summarized using mean and standard deviation for continuous variables and count and percent for categorical variables. To examine the associations of 25OHD and cytokines on breast cancer ER status (ER negative *vs.* ER positive), each analyte or cytokine ratio was dichotomized at the median (high *vs.* low) and examined in relation to ER status using unconditional logistic regression. Odds ratio (OR) and 95% confidence intervals (CI) were derived with adjustment for age at diagnosis, specimen storage time, season of blood draw, timing of blood draw in relation to receipt of treatment, and American Joint Committee on Cancer (AJCC) breast cancer stage. Further adjustment with other potential covariates, including breast cancer family history, body mass index, tumor grade, and smoking status did not significantly alter the estimates, and were thus not included. Correlation between levels of 25OHD and cytokines/ratios was tested using Spearman correlation with adjustment for the same set of covariates. To test whether the associations of 25OHD and cytokines on cancer ER status were independent from each other, mutual adjustment in the logistic regression models with the same covariates was performed. 

To examine the combinational associations of 25OHD and cytokines on breast cancer ER status, a composite variable between 25OHD and each cytokine/ratio with four levels was created (high 25OHD low cytokine, high 25OHD high cytokine, low 25OHD low cytokine, and low 25OHD high cytokine) and examined in relation to ER status using similar logistic regression models as described above, with women at high 25OHD and low cytokine levels as the reference group (presumably at the lowest risk based on previous analyses of the main effects). For the four Th1 cytokine to IL5 ratios, where IL5 was used as a denominator (IL12p70/IL5, IFNγ/IL5, CXCL10/IL5, and TNFα/IL5), the high and low risk categories were reversed as the low level of each ratio was associated with increased risk. Additive interactions were estimated using the relative excess risk due to interaction (RERI = OR_11_ − OR_10_ − OR_01_ + 1), where OR_11_, OR_10_ and OR_01_ were odds ratios associated with low 25OHD high cytokine (highest risk group), low 25OHD low cytokine (sub-high risk group), and high 25OHD and high cytokine (sub-high risk group), respectively. Confidence intervals and *p*-values for RERI were computed by published SAS program by Lundberg and Andersson [14,15]. An RERI above 0 would indicate a synergistic association and an RERI below 0 would indicate an antagonistic association. Multiplicative interactions were tested by adding the cross product terms between the binary 25OHD level and each of the binary cytokine/ratio to the logistic regression model, and the Wald test was used to estimate the significance of multiple interactions. A *p*-value <0.05 was considered statistically significant for main effects. Given the relatively limited sample size, a *p*-value for interaction <0.20 was considered statistically significant. All analyses were performed in SAS 9.3.

## 3. Results

Descriptive characteristics of the 490 patients with invasive breast cancer are summarized in Table 1 for all patients or by cancer ER status. The average age of diagnosis was 56 years, with approximately 41% of women diagnosed before menopause. Close to 70% of women were overweight or obese (BMI ≥25 kg/m^2^) and only 19% of women were considered vitamin D sufficient (serum 25ODH ≥30 ng/mL). Most of the women had early stage breast cancer (stage I-IIIA), 63% of tumors had high histological grade, and 23% were ER negative. 

**Table 1:** Descriptive characteristics of breast cancer patients from the Data Bank and Biorepository (DBBR) overall and by estrogen receptor status.

Characteristics	All patients (n = 490)	ER-negative (n = 113)	ER-positive (n = 370)
Age at diagnosis, years, mean ± sd	56.4 ± 12.9	52.3 ± 12.7	57.6 ± 12.8
Menopausal status, N (%)			
Premenopausal	200 (40.8)	53 (46.9)	145 (39.2)
Postmenopausal	290 (59.2)	60 (53.1)	225 (60.8)
Body mass index			
Normal (<25 kg/m^2^)	145 (30.3)	34 (30.3)	111 (30.9)
Overweight (25–29.9 kg/m^2^)	166 (34.7)	46 (41.1)	118 (32.9)
Obese (≥ 30 kg/m^2^)	168 (35.0)	32 (28.6)	130 (36.2)
Vitamin D status			
Sufficient (≥30.0 ng/ml)	93 (19.0)	22 (19.5)	70 (18.9)
Insufficient (20–29.9 ng/ml)	195 (39.8)	41 (36.3)	149 (40.3)
Deficient (<20 ng/ml)	202 (41.2)	50 (44.3)	151 (40.8)
Stage, N (%)			
I	293 (59.8)	50 (44.2)	237 (64.1)
II/IIIA	177 (36.1)	58 (51.3)	119 (32.2)
IIIB/IIIC/IV	20 (4.1)	5 (4.4)	14 (3.8)
Histological grade, N (%)			
I	45 (9.3)	1 (0.9)	42 (11.5)
II	129 (26.7)	15 (13.5)	114 (31.1)
III	310 (64.0)	95 (85.6)	210 (57.4)

For some variables the count and percent do not add up to the total due to missing data. Abbreviation: sd, standard deviation; ER, estrogen receptor.

### 3.1. Correlations between Levels of 25OHD and Cytokines/Ratios

Spearman partial correlations between serum levels of 25OHD and plasma levels of selected cytokines and ratios with adjustment for covariates are shown in Table 2. There were moderate positive correlations among IL5, TNFα, and IFNα2, as well as with cytokine ratios containing these cytokines. However, there was no correlation of 25OHD with any of the cytokines or ratios. No correlation was found after stratification by either menopausal status or ER status (data not shown).

### 3.2. Main Effects of 25OHD and Cytokines/Ratios on Cancer ER Status

As we previously reported [11,12] and is shown in Table 3, among premenopausal breast cancer patients, low serum levels of 25OHD were associated with increased odds of ER negative vs. ER positive cancer, while high plasma levels of IL5, IFNα2 and TNFα were associated with increased odds of ER negative in comparison to ER positive cancer. In addition, the higher ratios of IFNα2 to Th2 cytokines, including IFNα2/IL4, IFNα2/IL10, IFNα2/CCL2, IFNα2/CCL7, IFNα2/CCL11, and IFNα2/TNFα, were associated with increased odds of ER negative *vs.* ER positive cancer. Lower ratios of Th1 cytokines to IL5, including IL12p70/IL5, IFNγ/IL5, CXCL10/IL5, and TNFα/IL5, were associated with higher odds of ER negative disease. To examine whether the associations of 25OHD and cytokines/ratios were independent from each other, we mutually adjusted for each other in the same multivariate models, and the associations remained essentially unchanged (data not shown). 

### 3.3. Combinational Associations of 25OHD and Cytokines/Ratios on ER Status in Premenopausal Breast Cancer Patients

Because the main effects of 25OHD and cytokines on ER status were found only in premenopausal patients, their combinational associations were analyzed in this subgroup only. The ORs and 95% CIs shown in Table 4 for each of the four groups consisted of high and low levels of 25OHD and cytokines/ratios, as well as RERI and *p*-values for additive and multiplicative interactions. The most notable synergistic association was between 25OHD and TNFα. Compared to the lowest risk group (high 25OHD and low TNFα levels), patients in the highest risk group (low 25OHD and high TNFα levels) had more than 7-fold increased odds of ER negative *vs.* ER positive cancer (OR = 7.32, 95% CI = 2.44–21.98). The estimated RERI was 5.46 (95% CI = −1.84–12.76); p for additive interaction was 0.14 and *p* for multiplicative interaction was 0.09. Similar synergistic associations were also found between 25OHD and IL5, as well as seven cytokine ratios, including IFNα2/IL4, IFNα2/IL10, IFNα2/CCL2, IFNα2/TNFα, IL12p70/IL5, IFNγ/IL5, CXCL10/IL5, and TNFα/IL5, although tests for additive or multiplicative interactions were not significant (Table 4). We also found evidence of antagonistic association between 25OHD and INFα2, as the OR in the highest risk group, 7.68 (95% CI = 2.25–26.23), was lower than the addition of the ORs of each factor alone, and the RERI was below 0 (RERI = −1.83, 95% CI = −9.33–5.67, *p* for additive interaction =0.63 and *p* for multiplicative interaction =0.10). Similar antagonistic associations were also found between 25OHD and IFNα2/CCL7 and IFNα2/CCL11.

**Table 2:** Spearman correlations of pretreatment levels of 25OHD with cytokines and ratios in women diagnosed with invasive breast cancer.

Variable	25OHD	IL5	IFNα2	TNFα	IFNα2/IL4	IFNα2/IL10	IFNα2/CCL2	IFNα2/CCL7	IFNα2/CCL11	IFNα2/TNFα		IL12p70/IL5	IFNγ/IL5	CXCL10/IL5
IL5	0.05													
IFNα2	0.08	0.40 ^***^												
TNFα	0.05	0.40 ^***^	0.33 ^***^											
IFNα2/IL4	−0.06	0.07	0.35 ^***^	0.11 ^*^										
IFNα2/IL10	0.04	0.23 ^***^	0.81 ^***^	0.22 ^***^	0.42 ^***^									
IFNα2/CCL2	0.09	0.38 ^***^	0.97 ^***^	0.28 ^***^	0.32 ^***^	0.78 ^***^								
IFNα2/CCL7	−0.03	0.12 ^**^	0.66 ^***^	0.04	0.41 ^***^	0.60 ^***^	0.63 ^***^							
IFNα2/CCL11	0.03	0.38 ^***^	0.96 ^***^	0.32 ^***^	0.33 ^***^	0.78 ^***^	0.94 ^***^	0.64 ^***^						
IFNα2/TNFα	0.06	0.22 ^***^	0.87 ^***^	−0.11 ^*^	0.32 ^***^	0.73 ^***^	0.87 ^***^	0.70 ^***^	0.84 ^***^					
IL12p70/IL5	0.01	−0.64 ^***^	0.00	0.00	0.06	0.07	0.00	−0.09	−0.01	−0.02				
IFNγ/IL5	0.03	−0.82 ^***^	−0.18 ^***^	−0.15 ^***^	0.03	−0.06	−0.18 ^***^	−0.14 ^**^	−0.18 ^***^	−0.12 ^**^		0.77 ^***^		
CXCL10/IL5	−0.07	−0.96 ^***^	−0.38 ^***^	−0.31 ^***^	−0.07	−0.24 ^***^	−0.38 ^***^	−0.12 ^**^	−0.36 ^***^	−0.24 ^***^		0.63 ^***^	0.81 ^***^	
TNFα/IL5	−0.04	−0.95 ^***^	−0.33 ^***^	−0.14 ^**^	−0.03	−0.18 ^***^	−0.33 ^***^	−0.12 ^**^	−0.31 ^***^	−0.28 ^***^		0.70 ^***^	0.85 ^***^	0.95 ^***^

Partial correlations adjusted for age at diagnosis, season, date of blood draw, timing of blood draw in relation to surgery and treatment, and tumor stage. *, *p* < 0.05; **, *p* < 0.01; ***, *p* < 0.001.

**Table 3:** Associations of blood levels of 25OHD and cytokines with odds of estrogen receptor (ER) negative breast cancer versus ER positive breast cancer.

Cytokine/Ratio	Level	All women			Premenopausal women			Postmenopausal women		
		# ER− *vs.* ER+	OR (95% CI)	*p* *	# ER− *vs.* ER+	OR (95% CI)	*p* *	# ER− *vs*. ER+	OR (95% CI)	*p* *
25OHD	High	52/188	1.00		23/85	1.00		29/103	1.00	
	Low	61/182	1.24 (0.80–1.92)	0.34	30/60	2.08 (1.05–4.11)	0.04	31/122	0.78 (0.42–1.45)	0.44
IL5	Low	53/187	1.00		20/87	1.00		33/100	1.00	
	High	60/183	1.25 (0.8–1.95)	0.32	33/58	3.09 (1.51–6.29)	0.002	27/125	0.7 (0.38–1.28)	0.25
IFNα2	Low	45/194	1.00		16/75	1.00		29/119	1.00	
	High	68/176	1.57 (1.01–2.44)	0.05	37/70	2.46 (1.22–4.95)	0.01	31/106	1.15 (0.62–2.11)	0.66
TNFα	Low	55/188	1.00		28/104	1.00		27/84	1.00	
	High	58/182	1.38 (0.87–2.17)	0.17	25/41	2.27 (1.14–4.51)	0.02	33/141	0.74 (0.4–1.37)	0.34
IFNα2/IL4	Low	46/195	1.00		21/81	1.00		25/114	1.00	
	High	67/175	1.54 (0.99–2.4)	0.06	32/64	1.99 (1.01–3.94)	0.05	35/111	1.19 (0.64–2.21)	0.59
IFNα2/IL10	Low	45/194	1.00		16/70	1.00		29/124	1.00	
	High	68/176	1.65 (1.06–2.58)	0.03	37/75	2.25 (1.11–4.54)	0.02	31/101	1.43 (0.78–2.62)	0.25
IFNα2/CCL2	Low	45/194	1.00		15/68	1.00		30/126	1.00	
	High	68/176	1.56 (0.99–2.44)	0.05	38/77	2.44 (1.17–5.09)	0.02	30/99	1.23 (0.67–2.26)	0.51
IFNα2/CCL7	Low	45/194	1.00		21/82	1.00		24/112	1.00	
	High	68/176	1.65 (1.06–2.57)	0.03	32/63	1.71 (0.87–3.37)	0.12	36/113	1.5 (0.81–2.78)	0.20
IFNα2/CCL11	Low	45/193	1.00		17/70	1.00		28/123	1.00	
	High	68/177	1.49 (0.95–2.32)	0.08	36/75	1.87 (0.94–3.74)	0.08	32/102	1.26 (0.69–2.32)	0.45
IFNα2/TNFα	Low	39/200	1.00		14/70	1.00		25/130	1.00	
	High	74/170	2.03 (1.29–3.2)	0.002	39/75	2.36 (1.14–4.9)	0.02	35/95	1.87 (1.01–3.46)	0.05
IL12p70/IL5	High	48/194	1.00		23/82	1.00		25/112	1.00	
	Low	65/176	1.54 (0.99–2.4)	0.06	30/63	1.83 (0.93–3.59)	0.08	35/113	1.39 (0.75–2.56)	0.30
IFNγ/IL5	High	55/188	1.00		24/85	1.00		31/103	1.00	
	Low	58/182	1.2 (0.77–1.87)	0.41	29/60	2.24 (1.12–4.5)	0.02	29/122	0.8 (0.44–1.46)	0.47
CXCL10/IL5	High	50/187	1.00		18/79	1.00		32/108	1.00	
	Low	63/183	1.27 (0.82–1.98)	0.29	35/66	2.28 (1.14–4.55)	0.02	28/117	0.84 (0.46–1.54)	0.57
TNFα/IL5	High	52/191	1.00		20/85	1.00		32/106	1.00	
	Low	61/179	1.34 (0.86–2.09)	0.20	33/60	2.82 (1.39–5.72)	0.004	28/119	0.83 (0.45–1.53)	0.56

* Odds ratios and p-values were adjusted for age at diagnosis, season of blood collection), date of blood draw, timing of blood draw in relation to surgery and treatment, and tumor stage.

**Table 4:** Combined effects between blood levels of 25OHD and cytokines on the estrogen receptor (ER) negative status in premenopausal breast cancer patients.

Cytokine/Ratio	Level	High 25OHD		Low 25OHD		RERI (95% CI)	P_additive	P_multiplicative
		# ER− *vs.* ER+	OR (95% CI) *	# ER− *vs.* ER+	OR (95% CI) *			
IL5	Low	9/52	1.00	14/33	2.97 (1.08–8.14)	2.43 (−2.8–7.67)	0.36	0.89
	High	11/35	1.98 (0.69–5.67)	19/25	6.38 (2.29–17.81)			
IFNα2	Low	4/44	1.00	19/41	5.41 (1.63–17.97)	−1.83 (−9.33-5.67)	0.63	0.10
	High	12/31	5.11 (1.43–18.22)	18/29	7.68 (2.25–26.23)			
TNFα	Low	13/54	1.00	10/31	1.42 (0.53–3.79)	5.46 (−1.84–12.76)	0.14	0.09
	High	15/50	1.44 (0.59–3.48)	15/10	7.32 (2.44–21.98)			
IFNα2/IL4	Low	11/51	1.00	12/34	1.71 (0.64–4.56)	1.05 (−1.77–3.88)	0.47	0.81
	High	10/30	1.77 (0.62–5.06)	20/30	3.54 (1.4–8.93)			
IFNα2/IL10	Low	6/41	1.00	17/44	2.86 (0.98–8.31)	0.55 (−3.59–4.69)	0.79	0.57
	High	10/29	2.78 (0.86–9)	20/31	5.19 (1.76–15.27)			
IFNα2/CCL2	Low	11/39	1.00	12/46	0.91 (0.35–2.42)	1.04 (−0.84–2.91)	0.28	0.40
	High	8/23	1.39 (0.45–4.26)	22/37	2.34 (0.94–5.83)			
IFNα2/CCL7	Low	6/50	1.00	17/35	3.6 (1.24–10.44)	−2.77 (−8.48–2.94)	0.34	0.06
	High	15/32	4.49 (1.51–13.34)	15/28	4.32 (1.42–13.12)			
IFNα2/CCL11	Low	6/42	1.00	17/43	2.69 (0.92–7.87)	−0.49 (−4.66–3.68)	0.82	0.37
	High	11/28	3.18 (0.99–10.17)	19/32	4.37 (1.5–12.76)			
IFNα2/TNFα	Low	5/42	1.00	18/43	3.06 (0.99–9.45)	0.53 (−3.87–4.93)	0.81	0.53
	High	9/28	2.85 (0.83–9.8)	21/32	5.43 (1.77–16.62)			
IL12p70/IL5	High	11/49	1.00	12/36	1.63 (0.61–4.34)	1.26 (−1.88–4.4)	0.43	0.78
	Low	12/33	1.86 (0.7–4.97)	18/27	3.75 (1.42–9.92)			
IFNγ/IL5	High	11/50	1.00	12/35	1.77 (0.66–4.77)	2.67 (−1.62–6.96)	0.22	0.42
	Low	13/35	1.64 (0.62–4.39)	17/25	5.09 (1.85–14.05)			
CXCL10/IL5	High	9/48	1.00	14/37	1.95 (0.73–5.23)	1.59 (−1.71–4.89)	0.34	0.75
	Low	9/31	1.73 (0.59–5.12)	21/29	4.28 (1.63–11.24)			
TNFα/IL5	High	10/52	1.00	13/33	2.27 (0.84–6.17)	2.4 (−1.78–6.58)	0.26	0.63
	Low	10/33	1.63 (0.57–4.62)	20/27	5.3 (2–14.08)			

* Odds ratios and *p*-values were adjusted for age at diagnosis, season of blood collection), date of blood draw, timing of blood draw in relation to surgery and treatment, and tumor stage.Abbreviations: ER, estrogen receptor; RERI, relative excess risk due to interaction; CI: confidence interval.

## 4. Discussion

In this study, we examined the combinational associations of serum levels of 25OHD with plasma levels of three cytokines, IFNα2, TNFα and IL5, as well as ten cytokine ratios, on ER status of breast cancer. There was no correlation between blood levels of 25OHD and any of the cytokines/ratios, and the main effects of 25OHD and cytokines on breast cancer ER status, primarily in premenopausal women, were independent of each other. However, when considered together, premenopausal women with low 25OHD and high TNFα had the highest likelihood of having ER negative breast cancer, which was higher than the addition of main effects of each. Similar synergistic associations were also found for IL5 and several cytokine ratios. On the contrary, there was some evidence for antagonistic associations between 25OHD and IFNα2 on ER status, which was also found for two cytokine ratios where IFNα2 was the numerator. These findings, although preliminary due to the limited sample size and retrospective measurement of the analytes, provide the first evidence for extensive interactions between vitamin D and immune factors, which may influence breast cancer risk and aggressive characteristics. 

The strongest synergistic association on breast cancer ER status was found between 25OHD and TNFα. As a pro-inflammatory cytokine from macrophages and tumor microenvironment, TNFα has been implicated in cancer promotion and progression [16], possibly by mediating epithelial-mesenchymal transition required for tumor invasion and angiogenesis [17,18,19], and has been investigated as a target for cancer therapy [20]. In breast tumor tissues, high expression of TNFα has been linked with ER negativity and cancer relapse [17,21]. While there are numerous studies of either TNFα or vitamin D in breast cancer, studies on their interactions are scarce. In cell culture and animal studies, vitamin D treatment was found to suppress the production of TNFα [22,23]. In a small randomized trial among patients with colorectal adenoma, 800 IU/day of vitamin D3 supplementation for over six months only non-significantly decreased TNFα by 13% [9]; while in two earlier small trials among patients with congestive heart failure or ambulatory adults, 2,000 IU/day of vitamin D_3_ had no effect on circulating levels of TNFα [8,24]. In our study, we did not find a correlation between blood levels of 25OHD and TNFα in breast cancer patients, which was consistent with the results of the two trials. Furthermore, we found that the effects of 25OHD and TNFα on breast cancer ER status were independent from each other. Nevertheless, the group of premenopausal patients with combined low 25OHD and high TNFα levels were at the highest odds of ER negative vs. ER positive breast cancer, much higher than the expected additive effects from each analyte. Given the independence of circulating levels and main effects between 25OHD and TNFα, this observed synergistic association implies separate but inter-connecting pathways underlying the roles of the two analytes on the etiology of breast cancer subtypes. The exact biological mechanisms for the synergy are unclear. 

Similarly to the case of TNFα, we also found synergistic associations between 25OHD and IL5 on breast cancer ER status in premenopausal patients. IL5 is produced by Th-2 lymphocytes and plays an important role in B-cell differentiation and eosinophilopoiesis [25]. IL5 has been linked with anti-tumor activities, specifically, tumor immune surveillance by eosinophils as demonstrated in lung and fibrosarcoma mice models [26,27]. In-depth studies of IL5 in breast cancer are lacking. In one small study of 105 breast cancer patients, IL5 expression could not be detected in tumor tissues [21], while in another study of 35 breast cancer patients and 24 women with benign breast diseases, serum IL5 level was significantly higher in cancer patients [28]. This finding, as well as the finding from our study that high IL5 level was associated with increased odds of ER negative *vs.* ER positive cancer, is inconsistent with the anti-tumor properties of IL5. It is possible that IL5-driven immune surveillance targets more strongly against ER positive cancer cells and thus the higher ratio of ER negative *vs.* ER positive odds in patients with high IL5 levels. Alternatively, this observation could be due to a stronger stimulation of IL5 response by ER negative cancer cells. Nonetheless, these observational findings need to be further investigated in prospective studies. 

Although vitamin D is known to enhance Th2 immune responses, the relationship between vitamin D and IL5 in human has been ambiguous. In two small trials among ambulatory or obese adults, vitamin D_3_ supplementation did not increase circulating IL5 concentrations [29,30], consistent with our finding of no correlation between 25OHD and IL5 levels in breast cancer patients. The potential biological mechanisms to explain the synergistic associations between 25OHD and IL5 on ER status in our study are unclear, but the observational finding may provide a new clue for future studies of the roles of vitamin D in mediating Th2 immune responses. 

In addition to the above synergistic associations, we also found some evidence of antagonistic associations between 25OHD and IFNα2, as well as IFNα2 to Th2 cytokine ratios, on breast cancer ER status in premenopausal patients. IFNα2 belongs to the large family of interferon alpha, which is Th1 cytokine in response primarily to viral infection. Deletion of *IFNA* gene has been observed in acute leukemia and glioma cases [31], and impaired interferon signaling was an immune deficiency common in human cancers, including breast cancer [32]. Although vitamin D generally suppresses Th1 immune responses, we did not find any correlation between circulating levels of 25OHD and IFNα2. The antagonistic associations on breast cancer ER status could possibly be due to over saturation of the deleterious effects resulting from low vitamin D and high IFNα2 levels. 

The synergistic associations between vitamin D and cytokines on breast cancer aggressive characteristics may have important implications to breast cancer prevention and prognosis. Both vitamin D and NSAIDs have been studied separately in relation to reduced breast cancer risk and superior prognosis [33,34,35,36,37,38,39]. Nevertheless, despite mounting evidence on extensive roles of vitamin D in regulating immune responses, these two potentially “actionable” cancer prevention agents have seldom been considered together. One previous study demonstrated a clear synergistic effect between calcitriol and NSAIDs on inhibiting the growth of prostate cancer cells *in vitro* [10]. Our study based on a cohort of breast cancer patients demonstrates for the first time that such synergistic effects may also exist in breast cancer, and calls for future prospective studies to evaluate the combined use of vitamin D and NSAIDs for cancer prevention and prognosis. 

A limitation of our study is that blood samples were drawn at the time of diagnosis, and may not necessarily reflect etiologic events but rather, could be the result of disease onset or progression. Although the average time from diagnosis to the time of blood draw was relatively short (27 days), and the majority of blood samples were collected before any treatment (92%), we still cannot exclude a possibility of reverse causal relationships between blood analytes and breast cancer aggressive characteristics. However, exclusion of the samples collected after the initiation of treatment had little impact on the results. Another limitation of our study is the relatively small number of patients included in the analysis, particularly in subgroup analyses among premenopausal women. As a result, none of the interaction tests reached statistical significance when multiple comparison error was considered. Due to the concern of multiple testing, we refrained from analyzing the interactions between 25OHD and all cytokines and their ratios examined in our previous study, but instead focused on those shown significant main effects. However, it is possible that significant interactions exist even without significant main effects. In our previous analyses of the main effects of 25OHD and cytokines, we examined both ER status and triple-negative subtype *vs.* luminal A subtype. However, when examining the combinational associations between 25ODH and cytokines, the numbers of triple-negative breast cancer patients were rather limited, especially after stratifying by menopausal status. Although associations for triple negative breast cancer were similar to those reported here for ER status, that women with low 25OHD and high cytokine were most likely to have triple negative breast cancer vs. luminal A subtype (data not shown), the confidence intervals of the ORs were very wide; thus, we reported only the results of ER status. 

## 5. Conclusions

In women diagnosed with invasive breast cancer, we found no correlation of serum 25OHD levels with plasma levels of cytokines/ratios, and associations of 25OHD and cytokines with ER status were independent of each other. When 25OHD and cytokines/ratios were combined in our analyses, synergistic associations on ER status were found between 25OHD and TNFα and IL5, as well as several cytokine ratios, in premenopausal breast cancer patients. Caveats of a limited sample size and measurement of blood levels 25OHD and cytokines at the time of diagnosis should be considered when interpreting our results. Our study provides the first evidence of potential interactions between 25OHD and the immune system in modulating breast cancer aggressive characteristics, which may warrant further investigation in a future large prospective study.

## References

[1] Baeke F., Takiishi T., Korf H., Gysemans C., Mathieu C. (2010). Vitamin D: Modulator of the immune system. Curr. Opin. Pharmacol..

[2] Mullin G.E., Dobs A. (2007). Vitamin D and its role in cancer and immunity: A prescription for sunlight. Nutr. Clin. Pract..

[3] Hewison M. (2012). An update on vitamin D and human immunity. Clin. Endocrinol..

[4] Pludowski P., Holick M.F., Pilz S., Wagner C.L., Hollis B.W., Grant W.B., Shoenfeld Y., Lerchbaum E., Llewellyn D.J., Kienreich K. (2013). Vitamin D effects on musculoskeletal health, immunity, autoimmunity, cardiovascular disease, cancer, fertility, pregnancy, dementia and mortality-A review of recent evidence. Autoimmun. Rev..

[5] Adams J.S., Hewison M. (2008). Unexpected actions of vitamin D: New perspectives on the regulation of innate and adaptive immunity. Nat. Clin. Pract. Endocrinol. Metab..

[6] Kriegel M.A., Manson J.E., Costenbader K.H. (2011). Does vitamin D affect risk of developing autoimmune disease? A systematic review. Semin. Arthritis. Rheum..

[7] Ramagopalan S.V., Heger A., Berlanga A.J., Maugeri N.J., Lincoln M.R., Burrell A., Handunnetthi L., Handel A.E., Disanto G., Orton S.M. (2010). A ChIP-seq defined genome-wide map of vitamin D receptor binding: Associations with disease and evolution. Genome Res..

[8] Schleithoff S.S., Zittermann A., Tenderich G., Berthold H.K., Stehle P., Koerfer R. (2006). Vitamin D supplementation improves cytokine profiles in patients with congestive heart failure: A double-blind, randomized, placebo-controlled trial. Am. J. Clin. Nutr..

[9] Hopkins M.H., Owen J., Ahearn T., Fedirko V., Flanders W.D., Jones D.P., Bostick R.M. (2011). Effects of supplemental vitamin D and calcium on biomarkers of inflammation in colorectal adenoma patients: A randomized, controlled clinical trial. Cancer Prev. Res..

[10] Moreno J., Krishnan A.V., Swami S., Nonn L., Peehl D.M., Feldman D. (2005). Regulation of prostaglandin metabolism by calcitriol attenuates growth stimulation in prostate cancer cells. Cancer Res..

[11] Yao S., Sucheston L.E., Millen A.E., Johnson C.S., Trump D.L., Nesline M.K., Davis W., Hong C.C., McCann S.E., Hwang H. (2011). Pretreatment serum concentrations of 25-hydroxyvitamin D and breast cancer prognostic characteristics: A case-control and a case-series study. PLoS One.

[12] Hong C.C., Yao S., McCann S.E., Dolnick R.Y., Wallace P.K., Gong Z., Quan L., Lee K.P., Evans S.S., Repasky E.A. (2013). Pretreatment levels of circulating Th1 and Th2 cytokines, and their ratios, are associated with ER-negative and triple negative breast cancers. Breast Cancer Res. Treat..

[13] Ambrosone C.B., Nesline M.K., Davis W. (2006). Establishing a cancer center data bank and biorepository for multidisciplinary research. Cancer Epidemiol. Biomarkers Prev..

[14] Lundberg M., Fredlund P., Hallqvist J., Diderichsen F.A. (1996). SAS program calculating three measures of interaction with confidence intervals. Epidemiology.

[15] Andersson T., Alfredsson L., Kallberg H., Zdravkovic S., Ahlbom A. (2005). Calculating measures of biological interaction. Eur. J. Epidemiol..

[16] Balkwill F. (2006). TNFα in promotion and progression of cancer. Cancer Metastasis Rev..

[17] Soria G., Ofri-Shahak M., Haas I., Yaal-Hahoshen N., Leider-Trejo L., Leibovich-Rivkin T., Weitzenfeld P., Meshel T., Shabtai E., Gutman M. (2011). Inflammatory mediators in breast cancer: coordinated expression of TNFα & IL-1β with CCL2 & CCL5 and effects on epithelial-to-mesenchymal transition. BMC Cancer.

[18] Li C.W., Xia W., Huo L., Lim S.O., Wu Y., Hsu J.L., Chao C.H., Yamaguchi H., Yang N.K., Ding Q. (2012). Epithelial-mesenchymal transition induced by TNF-α requires NF-κB-mediated transcriptional upregulation of Twist1. Cancer Res..

[19] Asiedu M.K., Ingle J.N., Behrens M.D., Radisky D.C., Knutson K.L. (2011). TGFβ/TNFα-mediated epithelial-mesenchymal transition generates breast cancer stem cells with a claudin-low phenotype. Cancer Res..

[20] Burton E.R., Libutti S.K. (2009). Targeting TNF-alpha for cancer therapy. J. Biol..

[21] Chavey C., Bibeau F., Gourgou-Bourgade S., Burlinchon S., Boissiere F., Laune D., Roques S., Lazennec G. (2007). Oestrogen receptor negative breast cancers exhibit high cytokine content. Breast Cancer Res..

[22] Zhang Y., Leung D.Y., Richers B.N., Liu Y., Remigio L.K., Riches D.W., Goleva E. (2012). Vitamin D inhibits monocyte/macrophage proinflammatory cytokine production by targeting MAPK phosphatase-1. J. Immunol..

[23] Zhu Y., Mahon B.D., Froicu M., Cantorna M.T. (2005). Calcium and 1α,25-dihydroxyvitamin D3 target the TNF-alpha pathway to suppress experimental inflammatory bowel disease. Eur. J. Immunol..

[24] Yusupov E., Li-Ng M., Pollack S., Yeh J.K., Mikhail M., Aloia J.F. (2010). Vitamin D and serum cytokines in a randomized clinical trial. Int. J. Endocrinol..

[25] Takatsu K., Nakajima H. (2008). IL-5 and eosinophilia. Curr. Opin. Immunol..

[26] Ikutani M., Yanagibashi T., Ogasawara M., Tsuneyama K., Yamamoto S., Hattori Y., Kouro T., Itakura A., Nagai Y., Takaki S. (2012). Identification of innate IL-5-producing cells and their role in lung eosinophil regulation and antitumor immunity. J. Immunol..

[27] Simson L., Ellyard J.I., Dent L.A., Matthaei K.I., Rothenberg M.E., Foster P.S., Smyth M.J., Parish C.R. (2007). Regulation of carcinogenesis by IL-5 and CCL11: A potential role for eosinophils in tumor immune surveillance. J. Immunol..

[28] Lyon D.E., McCain N.L., Walter J., Schubert C. (2008). Cytokine comparisons between women with breast cancer and women with a negative breast biopsy. Nurs. Res..

[29] Barker T., Martins T.B., Hill H.R., Kjeldsberg C.R., Henriksen V.T., Dixon B.M., Schneider E.D., Dern A., Weaver L.K. (2012). Different doses of supplemental vitamin D maintain interleukin-5 without altering skeletal muscle strength: A randomized, double-blind, placebo-controlled study in vitamin D sufficient adults. Nutr. Metab..

[30] Jorde R., Sneve M., Torjesen P.A., Figenschau Y., Goransson L.G., Omdal R. (2010). No effect of supplementation with cholecalciferol on cytokines and markers of inflammation in overweight and obese subjects. Cytokine.

[31] Colamonici O.R., Domanski P., Platanias L.C., Diaz M.O. (1992). Correlation between interferon (IFN) alpha resistance and deletion of the IFN α/β genes in acute leukemia cell lines suggests selection against the IFN system. Blood.

[32] Critchley-Thorne R.J., Simons D.L., Yan N., Miyahira A.K., Dirbas F.M., Johnson D.L., Swetter S.M., Carlson R.W., Fisher G.A., Koong A. (2009). Impaired interferon signaling is a common immune defect in human cancer. Proc. Natl. Acad. Sci. USA.

[33] Blair C.K., Sweeney C., Anderson K.E., Folsom A.R. (2007). NSAID use and survival after breast cancer diagnosis in post-menopausal women. Breast Cancer Res. Treat..

[34] Kwan M.L., Habel L.A., Slattery M.L., Caan B. (2007). NSAIDs and breast cancer recurrence in a prospective cohort study. Cancer Causes Control.

[35] Holmes M.D., Chen W.Y., Li L., Hertzmark E., Spiegelman D., Hankinson S.E. (2010). Aspirin intake and survival after breast cancer. J. Clin. Oncol..

[36] Takkouche B., Regueira-Mendez C., Etminan M. (2008). Breast cancer and use of nonsteroidal anti-inflammatory drugs: A meta-analysis. J. Natl. Cancer Inst..

[37] Goodwin P.J., Ennis M., Pritchard K.I., Koo J., Hood N. (2009). Prognostic effects of 25-hydroxyvitamin D levels in early breast cancer. J. Clin. Oncol..

[38] Vrieling A., Hein R., Abbas S., Schneeweiss A., Flesch-Janys D., Chang-Claude J. (2011). Serum 25-hydroxyvitamin D and postmenopausal breast cancer survival: A prospective patient cohort study. Breast Cancer Res..

[39] Piura E., Chapman J.W., Lipton A., Zhu L., Leitzel K., Wilson C.F., Pritchard K.I., Shepherd L., Pollak M.N. (2009). Serum 1-OH vitamin D (D) and prognosis of postmenopausal breast cancer (BC) patients: NCIC-CTG MA14 trial. J. Clin. Oncol..

